# Effect of virtual reality-based education on death literacy and health among family caregivers: an experimental study

**DOI:** 10.1007/s00520-026-11318-1

**Published:** 2026-10-12

**Authors:** Hsiao-Lan Yu, Sheng-Miauh Huang, Chun-Kai Fang, Chen-Jung Chen

**Affiliations:** 1 Nurse Practitioner Development & Management Center, MacKay Memorial Hospital, New Taipei, Taiwan; 2 Department of Nursing, Nursing and Management, MacKay Junior College of Medicine, Taipei, Taiwan; 3 Department of Nursing, National Yang Ming Chiao Tung University Hospital, Yi-Lan, Taiwan; 4 College of Nursing, National Yang Ming Chiao Tung University, No. 155, Sec. 2, Linong St. Beitou Dist., Taipei City, 112304 Taiwan; 5 Hospice and Palliative Care Center and Department of Psychiatry, MacKay Memorial Hospital, New Taipei, Taiwan; 6 Department of Nursing, MacKay Medical University, New Taipei City, Taiwan

**Keywords:** Caregiver, Death literacy, Education, End-of-life, Palliative care, Virtual reality

## Abstract

**Purpose:**

Growing evidence suggests that virtual reality (VR) can improve the physical and psychological health of patients with cancer; however, little is known about its effects on family caregivers of patients receiving palliative care. The aim of this study was to evaluate the effect of a VR-based education system on death literacy and health among family caregivers of patients at the end-of-life.

**Methods:**

This study was conducted using a quasi-experimental design. A total of 66 family caregivers were recruited from a medical center in northern Taiwan and assigned to control (traditional consultation) or experimental (VR-enhanced education) groups according to the time of construction of the VR system for death education. All participants were assessed using the Death Literacy Index (DLI) and the Chinese Health Questionnaire-12 (CHQ-12) before and after the intervention.

**Results:**

Significant improvements in total DLI and related subscales were observed in the VR group. Interaction analyses indicated greater benefits for caregivers with lower baseline death literacy levels. The Johnson–Neyman technique revealed that the conditional effect of VR-based education on improving death literacy becomes statistically significant when the pretest score of total DLI was lower than 117.94. The CHQ-12 scores did not change significantly following the intervention.

**Conclusion:**

VR-enhanced education demonstrates feasibility and preliminary efficacy in enhancing death literacy among family caregivers in palliative care settings, particularly for those with lower baseline death literacy. However, its long-term impact on psychological health warrants further longitudinal investigation.

## Introduction

Despite advances in medicine and technology, many cancer patients at the end of life require supportive care each year. Although early palliative care improves the quality of life, symptom intensity, and functional well-being in patients with advanced cancer [1], the quality of end-of-life care in Asia remains significantly influenced by legal and cultural factors [2]. Taiwan has enacted two major laws (the Hospice Palliative Care Act and the Patient Right to Autonomy Act) to expand palliative care services, but there remains potential for improvement in practical implementation [3]. Overcoming common barriers to end-of-life care for patients with terminal cancer in Taiwan requires fostering open dialogue about death and advancing public education [4]. One key issue is that family caregivers provide substantial care for patients with advanced cancer while simultaneously bearing an invisible care burden and unmet needs [5], especially regarding information needs and challenges in death preparation and treatment decision making [6].

Death literacy is defined as a system of knowledge and skills that enables individuals to access, understand, and utilize information about death, dying, and end-of-life care, along with the various options available [7]. Death literacy index (DLI) was developed by the Caring at End-of-Life research team at Western Sydney University. The DLI encompasses four interrelated domains: practical understanding related to end-of-life care and available resources; experiential learning derived from caregiving experiences and interpersonal interactions; factual knowledge regarding the medical, legal, and procedural aspects of end-of-life care; and community capacity reflecting awareness and engagement with social networks and community resources that support end-of-life care [7–9]. From a supportive and palliative care perspective, The DLI provides a framework to understand caregiver preparedness as a core element, shaping how family caregivers perceive, interpret, and respond to end-of-life situations [8]. Since 2023, six integrated care systems in East England have been promoting death literacy knowledge mobilization among non-clinical communities [10]; however, no articles exist regarding its effectiveness.

Previous studies have shown that interventions supporting family caregivers in end-of-life cancer care vary considerably. Most intervention studies were delivered face-to-face or by phone and indicated improvements in psychological and emotional well-being, daily functioning, and social interactions [11]. Virtual reality (VR) technology holds potential value in cancer treatment by enhancing patient’s and caregivers’ knowledge of treatment and alleviating symptoms [12–16]. VR-based interventions offer opportunities to simulate end-of-life scenarios in a structured and emotionally contained manner, allowing caregivers to engage with complex situations that are otherwise difficult to anticipate [17]. However, empirical evidence regarding the effect of VR-enhanced supportive care on improving death literacy and reducing psychological wellbeing among family caregivers of patients with advanced cancer remains limited. Therefore, the aim of this study was to develop VR education targeting death literacy and evaluate the effectiveness of VR-enhanced education, implemented in conjunction with traditional counseling (focused on end-of-life needs and care), in enhancing death literacy and alleviating symptoms among family caregivers. To address the study aims, we proposed two hypotheses. First, family caregivers who receive VR-enhanced education in addition to traditional face-to-face consultation (the experimental group) will demonstrate greater improvements in death literacy compared to those receiving traditional face-to-face consultation alone (the control group). Second, family caregivers in the experimental group will demonstrate greater improvements in health status compared to those in the control group.

## Methods

### Study design and setting

This study was conducted using a quasi-experimental pretest–posttest design. Participants were family caregivers of patients receiving hospital-based palliative care consultations who had not been enrolled in hospice care. They were recruited from a palliative care consultation service at a medical center in northern Taiwan. During the development of VR-based death education, eligible family caregivers were allocated to the control group. After the program was developed, family caregivers were allocated to the experimental group. All participants were placed in a quiet, private space for the survey.

### Participants

Participants were eligible if they (1) were **a**dult family members providing unpaid care, (2) were involved in the patient’s daily care or decision-making processes, and (3) could communicate in Chinese and complete the study procedures. Caregivers were excluded if they were paid or professional caregivers or if they had cognitive impairment or severe physical or psychological conditions that would preclude completion of the questionnaires or participation in the VR intervention, as judged by the clinical care team. None of the patients was enrolled in hospice care at the time of caregiver recruitment. Sample size calculations were based on an effect size of 0.32 and a power of 0.8 by using a 2-sided t test with an α level of 0.05. This agreed with the sample size of 30 patients per group or 60 patients in total. Considering a dropout rate of 10%, a total of at least 66 patients needed to be enrolled.

### Intervention

Family caregivers in the control group received traditional face-to-face consultations that focused on end-of-life needs and care. Those in the experimental group received VR-enhanced education after traditional consultations. To ensure standardization and minimize environmental distractions, the intervention was delivered individually (one-on-one) in a quiet, private room. The researcher prepared and calibrated all devices and tools prior to each participant’s entry. The three modules were completed sequentially, lasting approximately 25 min in total. The three modules of the simulated scenarios were grounded in the concept of death literacy [8] and discussions by our research team, incorporating practical, experiential, factual, and community-based knowledge. These VR modules were designed to provide an **i**mmersive and experiential supportive care experience focused on end-of-life situations commonly encountered by family caregivers of patients with advanced cancer. The research team developed scripts through brainstorming. To ensure the importance, relevance, and cultural appropriateness of the VR scripts, five experts with clinical palliative care or educational experience were invited to review the content. They included a palliative care physician, a specialist nurse, a clinical nurse, a social worker, and a spiritual care practitioner. A Likert scale ranging from “Strongly Disagree” (1 point) to “Strongly Agree” (4 points) was used to reflect expert opinions of simulated scenarios. Evaluation of the VR-based death education program showed high importance, relevance, and cultural appropriateness, with mean scores of 3.87 (± 0.14), 3.78 (± 0.16) and 3.82 (± 0.16), respectively. These simulated scenarios enable learners to step into the world of end-of-life preparation, providing family caregivers with first-hand insights into death-related issues. They enhance caregiving and communication skills, promote experiential learning, and encourage reflection on supportive actions within family and societal contexts. Each module includes a knowledge quiz game and a user-friendly solution guide.

This study employed Meta Quest 3, Insta360 X4, and high-performance development workstations to construct a solution featuring high mobility and advanced environmental awareness capabilities. A virtual nurse was created using 3D modeling and real-time motion capture. The system was deployed on an HTC Virti immersive learning platform. After establishing the VR platform, the System Usability Scale (SUS) was used to conduct user-end trial evaluations, inviting five family caregivers to operate the system and provide feedback. The mean SUS score was 79.5 (± 3.26), indicating high user satisfaction with the platform. VR-based education was delivered in a structured and emotionally contained manner, with support from one nursing staff member, as needed, during the intervention.

### Measures

The study variables included demographics (sex, age, education, marital status, family relationship, income status, religion, presence of chronic disease, and availability of alternative caregiving personnel) and death literacy. Baseline data were collected before death education. Follow-up data were collected after death education, focusing on death literacy and health.

### Death literacy

Death literacy was defined as an individual’s knowledge and ability regarding end-of-life and dying care information. Therefore, the 29-item DLI was adopted as the measurement tool, encompassing four dimensions: eight items on practical knowledge, five items on experiential knowledge, seven items on factual knowledge, and nine items on community knowledge [8]. The Chinese version of the DLI is a reliable and valid instrument for measuring death literacy, with a reported Cronbach’s alpha of 0.941 [18]. In the present study, the 29-item DLI demonstrated excellent internal consistency, with Cronbach’s alpha values of 0.912 at pretest and 0.955 at posttest. The scale uses a Likert scale ranging from strongly disagree (1 point) to strongly agree (5 points), reflecting caregivers’ levels of preparedness and knowledge regarding end-of-life patients. Higher scores indicate greater death literacy.

### Health

The Chinese health questionnaire-12 (CHQ-12) is a mental and physical health screening tool specifically designed for the Chinese community. It was first introduced by Cheng and Williams in 1986 and later revised by Cheng et al. in 1990 [19, 20]. The scale consists of 12 items, including somatic symptoms, anxiety and worry, social dysfunction, depression, and poor family relationship. Each item is scored based on four response options: “no change from usual,” “better than usual,” “slightly worse than usual,” or “much worse than usual.” A score of 0 corresponds to “no change from usual or “better than usual” and a score of 1 corresponds to “slightly worse than usual” or “much worse than usual.” The total score ranges from 0 to 12, with higher scores indicating poorer mental health status. The previous study in Taiwan have reported Cronbach’s alpha coefficients of 0.83–0.84 for the CHQ-12 [19]. In the present study, the instrument demonstrated good internal consistency, with Cronbach’s alpha of 0.851 at pretest and 0.886 at posttest.

### Data analysis

Data were analyzed using IBM SPSS Statistics version 26.0 (IBM Corp., Armonk, NY, USA). Descriptive statistics summarized demographic characteristics and study variables, with means and standard deviations reported for continuous variables and frequencies and percentages for categorical variables. The primary analysis was performed using analysis of covariance (ANCOVA). Baseline equivalence between the experimental and control groups was examined using independent t-tests for continuous variables and chi-square tests for categorical variables. Within-group changes from baseline to post-intervention were assessed using paired t-tests. Variables that differed significantly between groups at baseline were included as additional covariates. When the assumption of homogeneity of regression slopes failed, the Johnson–Neyman (J–N) procedure served as the primary alternative to ANCOVA [21]. The J–N technique was further applied to depict moderation effects when the interaction effect (group × pretest score) was significant. This technique identifies areas within the range of pretest scores (moderating variable) where the group effect on the outcome is statistically significant. Analyses were conducted using the PROCESS macro for SPSS version 3.3 [22].

### Ethical considerations

This study was approved by the Institutional Review Board of MacKay Memorial Hospital (IRB No. 24MMHIS340e). All participants were provided with detailed information regarding the study purpose and procedures, and written informed consent was obtained prior to participation. Participation was voluntary, and caregivers were informed that refusal to participate or withdrawal from the study would not affect the care provided to them or the patients.

## Results

### Sample characteristics

A total of 67 family caregivers met the inclusion criteria, including 34 in the experimental group and 33 in the control group. One participant in the experimental group declined to participate owing to lack of interest in VR education. Most family caregivers were female (65.2%), married (69.7%), and had immediate relationships with the patients (72.7%). No significant differences were found between groups in chronic disease-related variables (Table 1). However, significant differences were observed in economic status and availability alternative caregiving personnel (*p* < 0.05, Table 1).

**Table 1:** Demographic characteristics and homogeneity of participants (*N* = 66)

Variable	Total (*N* = 66) n (%)	Control (*n* = 33) n (%)	Experimental (*n* = 33) n (%)	χ^2^	*p*
Gender				1.67	.19
Male	23 (34.8)	14 (42.4)	9 (27.3)		
Female	43 (65.2)	19 (57.6)	24 (72.7)		
Age (years)				2.73	.61
26–35	6 (9.1)	3 (9.1)	3 (9.1)		
36–45	10 (15.2)	3 (9.1)	7 (21.2)		
46–55	16 (24.2)	10 (30.3)	6 (18.2)		
56–65	21 (31.8)	10 (30.3)	11 (33.3)		
≥ 66	13 (19.7)	7 (21.3)	6 (18.2)		
Education				1.65	.44
Junior high school	12 (18.2)	4 (12.1)	8 (24.2)		
Senior high school	50 (75.8)	27 (81.8)	23 (69.7)		
Graduate degree	4 (6.1)	2 (6.1)	2 (6.1)		
Marital status				0.00	1.00
Unmarried	14 (21.2)	7 (21.2)	7 (21.2)		
Married	46 (69.7)	23 (69.7)	23 (69.7)		
Divorced/Widowed	6 (9.1)	3 (9.1)	3 (9.1)		
Family relationship				1.22	.27
Immediate	48 (72.7)	22 (66.7)	26 (78.8)		
Non-immediate	18 (27.3)	11 (33.3)	7 (21.2)		
Income status				7.20	.03*
More than expenses	7 (10.6)	4 (12.1)	3 (9.1)		
Balanced	47 (71.2)	19 (57.6)	28 (84.8)		
Less than expenses	12 (18.2)	10 (30.3)	2 (6.1)		
Religion				0.33	.57
Yes	50 (75.8)	26 (78.8)	24 (72.7)		
No	16 (24.2)	7 (21.2)	9(27.3)		
Chronic disease				3.42	0.06
Yes	21(31.8%)	7(21.2%)	14(42.4%)		
No	45(68.2%)	26(78.8%)	19(57.6%)		
Alternative caregiving personnel				5.28	0.02*
Yes	50(75.8%)	21(63.6%)	29(87.9%)		
No	16(24.2%)	12(36.4%)	4(12.1%)		

Independent *t* tests and chi-square tests were used. *p* < 0.05 indicates statistical significance

### Main effects on death literacy and health

Table 2 shows changes in DLI and CHQ-12 scores before and after intervention. After controlling for baseline death literacy scores and relevant covariates (economic status and alternative caregiving personnel), ANCOVA revealed a significant group effect of the intervention on total DLI scores (F = 25.99, *p* < 0.01, partial η^2^ = 0.427) and all subscales (practical knowledge: f = 6.88, *p* < 0.05, partial η^2^ = 0.104; experiential knowledge: f = 16.17, *p* < 0.01, partial η^2^ = 0.215; factual knowledge: f = 18.49, *p* < 0.01, partial η^2^ = 0.239; community knowledge: f = 38.06, *p* < 0.01, partial η^2^ = 0.392) (Table 3). No significant group effect of the intervention was observed for total CHQ-12 (Table 3). Across all measures, pretest scores were significant predictors of posttest scores (Table 3).

**Table 2:** Within-group changes in death literacy and health (*n* = 66)

Outcome	Group	Pretest Mean ± SD	Posttest Mean ± SD	*t*	*p*
Total DLI	Control	85.79 ± 20.45	90.52 ± 23.80	−2.12	.42
	Experimental	90.64 ± 19.27	115.64 ± 12.56	−13.93	<.01**
Practical knowledge	Control	26.39 ± 4.90	26.06 ± 7.16	0.35	.73
	Experimental	28.30 ± 6.80	32.06 ± 4.58	−7.51	<.01**
Experiential knowledge	Control	19.58 ± 4.06	19.36 ± 4.05	0.54	.60
	Experimental	19.79 ± 3.44	23.18 ± 2.08	−8.22	<.01**
Factual knowledge	Control	22.52 ± 6.85	24.42 ± 6.25	−2.17	.04*
	Experimental	23.48 ± 4.81	30.21 ± 2.03	−11.21	<.01**
Community knowledge	Control	17.30 ± 11.08	20.67 ± 11.50	−2.99	.01*
	Experimental	19.06 ± 12.93	30.18 ± 7.61	−7.62	<.01**
**Total CHQ-12**	Control	8.06 ± 2.81	8.36 ± 3.46	−0.56	.58
	Experimental	3.61 ± 3.13	3.42 ± 2.83	1.98	<.06

Differences were examined using paired-samples t tests. CHQ-12 = Chinese Health Questionnaire-12; *DLI*, Death Literacy Index; *SD*, standard deviation

^*^ *p* <.05; ** *p* <.01

**Table 3:** Group and moderating effects on death literacy using the ANCOVA and J–N technique

Outcome	Source	*F*	*p*	η^2^	J–N Cutoff values
**Total DLI**	Pretest score	177.89	<.01**	.751	
	Group	25.99	<.01**	.427	
	Pretest × Group	9.84	<.01**	.306	117.94
Practical knowledge	Pretest score	88.96	<.01**	.601	
	Group	6.88	<.05*	.104	
	Pretest × Group	2.90	.094	.047	-
Experiential knowledge	Pretest score	103.76	<.01**	.638	
	Group	16.17	<.01**	.215	
	Pretest × Group	7.39	<.01**	.111	24.94
Factual knowledge	Pretest score	57.96	<.01**	.496	
	Group	18.49	<.01**	.239	
	Pretest × Group	5.89	<.05*	.091	30.95
Community knowledge	Pretest score	126.61	<.01**	.682	
	Group	38.06	<.01**	.392	
	Pretest × Group	11.81	<.01**	.167	29.26
**Total CHQ-12**	Pretest score	115.45	<.01**	.662	
	Group	2.37	.129	.039	
	Pretest × Group	1.02	.316	.017	-

*ANCOVA*, analysis of covariance; *DLI*, death literacy index, *J–N*, Johnson–Neyman technique. Pretest scores were entered as covariates. Effect sizes are reported as partial eta squared (η^2^). Interaction terms (Pretest × Group) indicate whether the group effect varied according to baseline death literacy. Adjusted variables included income status and alternative caregiving personnel. * *p* <.05. ** *p* <.01

### Conditional effects on death literacy

Analysis of group-by-baseline interaction indicated significant effects, suggesting that intervention effectiveness varied according to caregivers’ baseline levels of total DLI and the experiential, factual, and community knowledge subscales. Finally, J–N analysis identified specific ranges of pretest scores encompassing the overall DLI and these three subscales, indicating that intervention effectiveness may vary under different pretest score conditions. VR-based education was associated with significantly greater improvements: total DLI score below 117.94 (ΔR^2^ = 0.03, F = 10.30, *p* < 0.01), experiential knowledge score below 24.94 (ΔR^2^ = 0.04, F = 9.91, *p* < 0.01), factual knowledge score below 30.95 (ΔR^2^ = 0.03, F = 4.44, *p* = 0.04), or community knowledge score below 29.26 (ΔR^2^ = 0.05, F = 11.70, *p* = 0.01) (Table 3).

## Discussion

Death literacy education is required to help caregivers access, understand, and make informed choices regarding end-of-life and death care options [8]. VR-based interventions can effectively convey complex information through interaction and exploration of simulated scenarios [15, 16]. To our knowledge, no studies have been conducted to develop VR-based applications for death literacy among caregivers of patients with cancer. Our research team developed an expert-validated VR tool and platform for death literacy, which has received high usability ratings in Taiwan. Future studies should evaluate its effectiveness in other populations.

Evidence suggests that VR has been widely used to support physical, emotional, and functional health in patients with cancer [13]. Our findings showed that VR-based death literacy education significantly improved overall death literacy and its subscales. Additionally, the J–N analysis provided specific data that more effectively identified the target population. This finding implies that VR-based death literacy education offers powerful supplementary benefits for learners with weak foundations. For family members unfamiliar with end-of-life care, VR can rapidly bridge knowledge gaps. Clinically, as the use of VR devices and health education sessions is typically time-limited, it is recommended that healthcare teams utilize the DLI as a screening tool prior to intervention. Family members with pre-intervention scores below 117.94 for total DLI, 24.94 for experiential knowledge, 30.95 for factual knowledge, and 29.26 for community knowledge should be prioritized for VR-based death education.

In the present study, pre-intervention DLI scores suggest that Taiwanese family caregivers had a neutral level of death literacy, indicating limited confidence in their knowledge and skills regarding death. Compared with other survey studies, Australian or Chinese populations have shown higher levels of confidence [8, 23]. The knowledge distribution in this study showed that community knowledge scored the lowest among the DLI domains, with responses averaging between “strongly disagree” and “disagree.” This aligns with earlier research indicating that most families are unfamiliar with available resources, legal options, and social support systems [9]. It also reflects a cultural tendency in Taiwanese society to view death as taboo, making related topics difficult to discuss openly [24]. Whether this relates to a state of estrangement in the relationship between patients with cancer and their families warrants further investigation [23]. Future research should continue refining VR-based educational content by integrating more local case studies and legal resources, potentially enhancing end-of-life literacy and health promotion outcomes among families of palliative patients in Taiwan.

In our pre-intervention assessment of health status, the control group exhibited poorer health than the experimental group. Pre-intervention health status was the only significant predictor of post-intervention scores. Although VR-based education failed to significantly improve family caregivers’ health status, it did not cause health deterioration. A slight improvement in health scores was observed among caregivers in the experimental group, while the control group showed a slight decline. Other studies have indicated that VR-based education improves family members’ mental health [2, 16]. Our findings diverge from those of previous studies, potentially owing to differences in study design (data collected only one-hour post-intervention) and subjects (caregivers of hospice patients). This palliative care phase coincides with rapid deterioration in patient health, placing caregivers under heightened stress, increased burden, and more intensive caregiving demands [25–28]. Although health differences between groups were not statistically significant, future studies should adopt longitudinal designs to track long-term health changes.

This study has several limitations. First, the quasi-experimental design and single-center setting may limit the generalizability of the findings. Second, VR-based education did not yield significant improvements in participants whose pretest scores exceeded the threshold. This result indicates that simple simulation scenarios may no longer meet the needs of family caregivers who already possess high levels of death literacy. Third, outcomes were assessed over a relatively short follow-up period, leaving long-term effects unknown. Finally, health and death literacy were measured using self-reported instruments, which may be subject to response bias. Future research using randomized designs, extended follow-up periods, and multicenter samples is warranted to further evaluate the effectiveness and sustainability of VR-enhanced supportive education.

## Conclusions

This study suggests that integrating VR-assisted education with traditional face-to-face counseling can enhance death literacy among family caregivers of palliative care patients, particularly in those with lower baseline readiness. Furthermore, specific baseline DLI thresholds were identified to help clinical teams target potential beneficiaries more effectively. Given the limited familiarity with community resources observed in our sample, future research should refine VR-based educational programs. Integrating localized cases and legal resources into these virtual environments may further enhance the end-of-life knowledge and skills of family caregivers**.**

## Data Availability

The data that support the findings of this study are not publicly available due to participant confidentiality but are available from the corresponding author upon reasonable request.
